# Bilateral facial nerve palsy complicating Kawasaki disease: A case report and literature review

**DOI:** 10.1097/MD.0000000000039389

**Published:** 2024-08-16

**Authors:** Rong Ou, Zhongyou Tan, Ling Liu

**Affiliations:** a Department of Pediatrics, Chongqing University Three Gorges Hospital, Chongqing, China; b School of Medicine, Chongqing University, Chongqing, China.

**Keywords:** coronary artery aneurysm, coronary artery lesions, facial nerve palsy, Kawasaki disease

## Abstract

**Rationale:**

Kawasaki disease (KD) manifests as an acute, self-limited vasculitis disease that constitutes the primary cause of acquired heart disease in children under 5 years of age. Facial nerve palsy (FNP) is a rare complication associated with coronary artery lesions (CALs) in patients with KD. Patients with KD and FNP usually present atypically, leading to a delayed diagnosis and treatment of KD.

**Patient concerns:**

A 4-month-old boy with fever, left FNP and bilateral conjunctival injection with spontaneous resolution, was admitted to the hospital, received a short course of intravenous dexamethasone, and experienced rapid FNP recovery on the first admission. The patient experienced a resurgence of fever, bilateral conjunctival injection, and right FNP, which led to readmission. Physical examination revealed redness at the site of Bacillus Calmette-Guérin inoculation, reddening of lips, and desquamation of the distal extremities. Echocardiography revealed right-sided CALs.

**Diagnoses:**

The patient initially missed KD on the first admission, and was later diagnosed with complete KD with FNP on the second admission.

**Interventions and outcomes:**

After a short course of intravenous dexamethasone, the left FNP resolved quickly. However, right FNP recurred after corticosteroids withdrawal. Meanwhile, more typical symptoms were observed, and KD was diagnosed. Treatment ensued with intravenous immunoglobulin (IVIG), aspirin, and dexamethasone. The patient achieved rapid remission, without recurrence. Echocardiography continued to show normal findings during 1-year follow-up after discharge.

**Lessons:**

The clinical symptoms of FNP complicating KD in children are atypical and can easily lead to delayed diagnosis and treatment. FNP in patients with KD may serve as a risk factor for CALs, which are more challenging to resolve than the FNP itself. Adding corticosteroids to IVIG may be recommended to reduce IVIG resistance, decrease the risk of developing CALs, and alleviate CALs.

## 1. Introduction

Kawasaki disease (KD), also known as mucocutaneous lymph node syndrome, manifests as an acute, self-limited vasculitis disease affecting various systems, including the skin, digestive tract, bones, respiratory tract, and nervous system. Neurological involvement is observed in 1% to 30% of patients with KD, and facial nerve palsy (FNP) is a rare complication, with an incidence of 0.06% reported by Liu et al.^[1]^ Patients with KD and FNP usually present atypically, leading to delayed diagnosis and treatment, and it is notably associated with coronary artery lesions (CALs). We report the case of a 4-month-old boy who presented with bilateral FNP and a right main coronary artery aneurysm (CAA) during the subacute phase of complete KD, the diagnosis of which was delayed until day 18 of illness.

## 2. Case report

A 4-month-old boy was admitted to a community hospital due to a 2-day history of fever. Cefathiamidine rendered him afebrile within 2 days. During the hospital stay, the patient manifested bilateral conjunctival injection and scattered vesicles in the distal extremities. On day 8 of the illness, he manifested right-sided deviation of the labial commissure and incomplete closure of the left eye and was referred to our hospital. Upon admission, he remained afebrile, and the bilateral conjunctival injection resolved. Positive laboratory findings indicated leukocytosis (17.55 × 10^9^/L), anemia (96 g/L), thrombocytosis (611 × 10^9^/L), elevated C-reactive protein (CRP) level (28.9 mg/L), and reduced sodium concentration (132 mmol/L). Brain magnetic resonance imaging (MRI) revealed no abnormalities. Neurological assessment confirmed left peripheral FNP without additional neurological deficits. Unfortunately, due to the incomplete features and early spontaneous fever remission that was likely caused by antibiotic treatment, his doctor overlooked the possibility of KD. The patient received a short course of intravenous dexamethasone at 0.2 mg/kg/day for 6 days. His temperature remained normal during the hospital stay. A routine blood test indicated normalization of the CRP level and elevated leukocytes (19.58 × 10^9^/L), hemoglobin (101 g/L), and platelets (898 × 10^9^/L) counts on the 5th day of hospitalization. His left FNP was not visible on the 7th day of hospitalization; so he was discharged and administered oral prednisone at 5 mg/day for 2 days.

**Figure 1.**
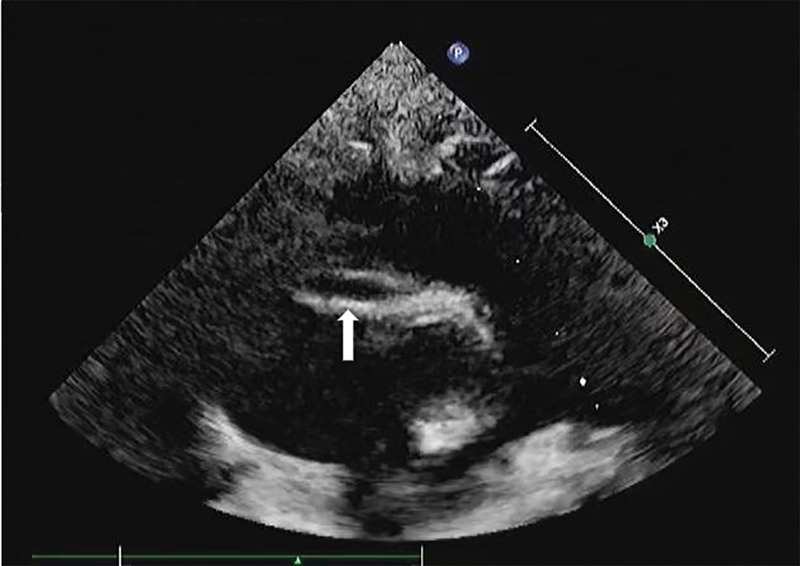
The echocardiographic image showing the right main coronary artery aneurysm (2.7 mm in diameter; *Z* score 4.06) (the arrow).

The patient experienced resurgence of fever and bilateral conjunctival injection on day 16 of the illness. Notably, on day 17 of the illness, the patient demonstrated an inability to close his right eye and exhibited a drooping mouth on the right side, leading to a diagnosis of right peripheral FNP. Consequently, he was readmitted to our hospital. Physical examination revealed redness at the site of Bacillus Calmette-Guérin (BCG) inoculation, reddening of lips and desquamation of the distal extremities without rash and palpable cervical lymph nodes. Laboratory findings indicated leukocytosis (22.15 × 10^9^/L), normal hemoglobin level (118 g/L), thrombocytosis (688 × 10^9^/L), elevated CRP level (117.7 mg/L), elevated erythrocyte sedimentation rate (49 mm/h), elevated procalcitonin (1.01 ng/mL), and decreased sodium concentration (132 mmol/L). Parameters such as aminotransferase, albumin, and urine leukocyte levels were within normal ranges. Echocardiography, conducted on the 2nd day of hospitalization (day 18 of illness), revealed the presence of a right main CAA (2.7 mm in diameter; *Z* score 4.06) (Fig. 1). Dexamethasone was administered at 0.2 mg/kg/day for 7 days on the first day of hospitalization. A diagnosis of complete KD was established, and treatment with intravenous immunoglobulin (IVIG) at a dosage of 2 g/kg and high-dose aspirin was initiated on the 3rd day of hospitalization. Rapid resolution of the fever and right FNP was observed on the 4th day of hospitalization. Subsequent echocardiography indicated normal coronary arteries, and normalization of the CRP level and white blood cell count occurred on the 7th day of hospitalization. Echocardiography continued to show normal findings during 1-year follow-up after discharge.

## 3. Systemic literature review

A search for patients aged 0 to 18 years with FNP and KD in database of PubMed, China How Net, Wan Fang, and VIP was performed for all years in April 2024. The following keywords were used: “neurological complication” or “neurological involvement” or “facial nerve palsy” or “facial nerve paralysis” or “facial palsy” or “facial paralysis” or “Bell palsy” and “Kawasaki disease.” Thirty seven worldwide articles were retrieved, including 1 Italian article, 1 French article, 1 Chinese article, 4 Japanese articles and 30 English articles. Finally, 56 pediatric patients with KD and FNP were identified.

## 4. Discussion

Details of the 56 well-described patients and of the patient reported herein with peripheral FNP and KD are shown in Table 1.^[2–38]^ Age ranged from 2 to 156 months, with a median age of 8 months and an average age of 14.7 months. Of the patients, 92.9% were aged ≤ 2 years and 66.1% were aged < 12 months. The female-to-male ratio was 1.24:1. The left side was involved in 60.7% (34/56) and the right side in 30.4% (17/56). Bilateral FNP was observed in only 5 patients. The median onset time of FNP was 16 days (range: 6–29 days). FNP (44.9%) mainly occurred in the second week. About one third of the population experienced FNP during the subacute phase (the second fever or afebrile period). Our literature analysis was similar to that of Stowe.^[26]^ Our patient had an early onset and rarely manifested bilateral FNP, which was only reported in previous 4 literatures.^[20,31,32,35]^ In our patient, delayed IVIG treatment (on day 19 of illness) may contribute to the recurrent FNP. This situation was also observed in 3 cases.^[20,31,32]^ However, many patients with KD with unilateral FNP do not use IVIG.^[26]^ Bilateral FNP is likely to be an occasional phenomenon.

**Table 1:** Characteristics of patients with Kawasaki disease and facial nerve palsy.

Literature	Case number	Age (mo)	Sex	Site of FNP	FNP onset (day of illness)	Time of IVIG (day of illness)	FNP onset after/before IVIG	Coronary involvement	Coronary involvement onset of illness	FNP before/after coronary involvement	iKD	CSF findings	Therapy	IVIG Resistance	Recovery of FNP	Recovery of Coronary Involvement
Murayama^[2]^	1	13	M	L	17	¯	¯	Bilateral CAA	¯	¯	¯	¯	¯	¯	Until death	Death by coronary embolism
Nakane et al^[3]^	1	7	M	L	26	>12	¯	None	None	None	¯	Normal	¯	¯	37 d	None
Amano and Hazama^[4]^	1	13	M	¯	¯	7	¯	¯	¯	¯	¯	WBC 123/mm^3^, normal protein and glucose	¯	¯	Until death	¯
Terasawa et al^[5]^	4	14; 9; 9; 6	4 F	2 R; 2 L	22; 10; 7; 18	¯	¯	Case 3: present; Case 1, 2, and 4: none	¯	¯	Case 1: no; Case 2, 3 and 4: not reported	Case1: WBC 21/mm^3^ (100% mononuclear cells), protein 0.58 g/L, sugar 0.46 g/L; Case 4: WBC 3/mm^3^(100% mononuclear cells), protein 0.13 g/L, sugar 0.59 g/L; Case 2 and 3: not done	Case 1: ASA; Case 2, 3, and 4: not reported	¯	Complete recovery at 2 wks, 10 d, 2 mo, and 3 wks, respectively	¯
Aso^[6]^	1	11	M	L	18	¯	¯	Bilateral CAA	¯	¯	¯	WBC 60/mm^3^ (85% mononuclear leukocytes, 15% polymorphonuclear leukocytes)	¯	¯	20 d	¯
Tamai et al^[7]^	4	15; 17; 8; 6	2 F; 2 M	2 R; 2 L	16; 16; 14;11	¯	¯	Bilateral CAA	¯	¯	¯	¯	¯	¯	13; 12; 16; 28 d	¯
Sabatino et al^[8]^	1	13	F	L	12	¯	¯	Bilateral CAA	¯	¯	¯	¯	¯	¯	16 d	¯
Hattori et al^[9]^	2	6; 9	2 F	1 L; 1 R	16; 19	None	None	None	None	None	¯	1 Normal; 1 Not done	ASA	¯	Complete recovery after 2 mo	None
Kleiman and Passo^[10]^	1	3	F	L	12	None	None	Bilateral CAA	18 d	Before	Yes	Normal in the first examination; WBC 26/mm^3^ (1% mononuclear leukocytes, 99% polymorphonuclear leukocytes), protein 0.31 g/L, sugar 0.58 g/L in the second examination after FNP	Digoxin, ASA, furosemide	¯	Full recovery after 7 d of FNP onset	Persistent large CAA after 4 mo
Gallagher^[11]^	1	10	F	R	10	None	None	None	None	None	No	Normal in the first examination; WBC 10/mm^3^ (83% lymphocytes, 3% segmented neutrophils, 14% monocytes), normal CSF chemistries in the second examination after FNP	ASA	¯	3 mo	None
Park et al^[12]^	1	7	F	R	17	>10	After	None	None	None	No	WBC 37/mm^3^ (96% mononuclear cells), protein 0.58 g/L, sugar 0.49 g/L, negative culture	IVIG, ASA	No	Full recovery within 40 d after illness	None
Bushara et al^[13]^	1	3	M	L	22	22	Before	Bilateral CAA (left 6.4 mm, right 6 mm)	21 d	After	No	WBC 7/mm^3^, protein 0.43 g/L, sugar 0.44 g/L, negative culture	IVIG, ASA, heparin	No	Complete resolution within 36 h of IVIG treatment	Gradual reduction of the size of CAAs at 12 mo follow-up
McDonald et al^[14]^	1	3.5	M	L	11	8	After	Aneurysms of the left anterior descending and circumflex coronary arteries	17 d	Before	Yes	Normal	¯	¯	Recovery	¯
Poon et al^[15]^	1	24	M	R	17	<10	After	None	None	None	No	¯	IVIG, ASA, dipyridamole	No	Full recovery within 1 mo	None
Biezeveld et al^[16]^	1	156	F	L	12	>10	After	Right CAA (5.0 mm)	In the first week	After	No	¯	IVIG, ASA	No	Complete resolution after 10 d	Recovery on day 11 of illness
Larralde et al^[17]^	1	5	M	R	> 11	27	Before	Right CAA and fusiform enlargement of the left CA	In the second week	After	No	¯	IVIG, ASA	No	Complete resolution over the next 6 wks	Recovery within 6 mo
Wright et al^[18]^	2	3; 7	2 M	2 L	25; -	> 9; none	Before; none	Case1: “beaded” appearance of the right CA, ectasia of proximal right CA (3.7 mm; *Z* score 7.93) and left anterior descending CA (2.3 mm; *Z* score 4.20), mild mitral valve incompetence; Case 2: giant bilateral CAA and axillary artery aneurysm	28 d; 20 mo	Before	1 No; 1 Yes	1 Negative culture; 1 Not done	Case 1: IVIG, ASA; Case 2: prednisolone, ASA, warfarin	Case 1: no	Case 1: resolution within 2 d; Case 2: a few days after treatment	Case1: marked improvement with minimal residual dilatation of the right CA (2 mm) and slight ectasia of the other CAs after 6 wks; Case 2: not reported
Li et al^[19]^	2	14; 11	1 M; 1 F	1 L; 1 R	17; >10	10; >10	After; Before	Case1: mild right (3.8 mm) and left (3.7 mm) CA dilatation, giant right (7.4 mm) and left (7.8 mm) CAA after recurrence of the fever; Case 2: right CA dilatation (4.3 mm)	In the second week; >10 d	Case 1: after; Case 2: before	No	¯	Case 1: IVIG (2 doses), ASA, dipyridamole; Case 2: IVIG, ASA	Case 1: yes; Case 2: no	Full recovery within 1 mo and 2 mo, respectively	Case1: 3.9 mm (right) and 3.3 mm (left) after 6 yrs; Case 2: resolution after 7 mo
Lim et al^[20]^	1	72	M	Bilateral	R 10; L 12	>14	R: before; L: after	Aneurysm of the left and right CAs, left anterior descending CA, and left circumflex CA; giant left anterior descending CAA	8 d	After	Yes	¯	IVIG, ASA, warfarin, prednisolone	Yes	Complete resolution：left, 23 d; right, 82 d	¯
Kaur et al^[21]^	1	2	F	L	12	>14	Before	Dilatation of right (8 mm) and left (6 mm) CAs and left anterior descending (8 mm) CA	>14 d	Before	Yes	WBC 30/mm^3^ (35% mononuclear cells), protein 0.71 g/L, sugar 0.63 g/L, negative culture	IVIG, ASA	No	Resolution within 2 d after IVIG treatment	¯
Alves et al^[22]^	1	¯	¯	L	26	¯	¯	Small left CAA	¯	¯	¯	¯	IVIG	¯	Improvement within 30 d	¯
Kocabaş et al^[23]^	1	8	F	L	<12	12	Before	Diffuse dilatation of all coronary arteries and aneurysm of left anterior descending and proximal right coronary artery with minimal pericardial fluid	14 d	Before	Yes	¯	IVIG, ASA, subcutaneous enoxaparin, warfarin	No	Resolution within 48 h after IVIG treatment	Three saccular aneurysmal dilatations of 9, 4.6, and 4.2 mm in diameter on RCA in the second month of the disease
Khubchandani and Dhanrajani ^[24]^	1	36	F	R	27	23	After	Diffuse dilatation of all coronary arteries and aneurysm of left anterior descending and proximal right coronary artery with minimal pericardial fluid	23 d	After	Yes	¯	IVIG	Yes	Full recovery after 3 wks	¯
Delafay et al^[25]^	1	14	F	L	¯	In the second week	Before	Hypoecho (3 mm in diameter) on the CA	In the second week	Before	Yes	WBC 15/mm^3^ (lymphocytic dominance), protein 0.15 g/L	IVIG, ASA	No	Full recovery after 20 d	¯
Stowe^[26]^	1	15	F	L	6	8	Before	Right CA dilatation (2.7 mm; *Z* score 2.46)	5 d	After	Yes	¯	IVIG, ASA, methylprednisolone	No	Full recovery after 5 d	No visualize coronary aneurysm at 1 and 3 mo
Chen et al^[27]^	3	4; 13; 15	1 M; 2 F	2 R; 1 L	10; 11; 16	>10	1 After; 2 Before	Dilatation of bilateral CAs	¯	¯	2 No; 1 Yes	Normal	IVIG, ASA, dexamethasone	No	Improvement after 1 wk	Case1: left CAA on day 11 of illness; Case 3: Right CAA on day 20 of illness
Orgun et al^[28]^	1	5	M	L	10	19	Before	Aneurysms of the left (3.8 mm; *Z* score 6.2) and right (3 mm; *Z* score 4.5) main CAs, left anterior descending CA A (2.6 mm; *Z* score 4.1), left circumflex CA A (2.5 mm; *Z* score 3.8)	¯	Before	No	WBC 15/mm^3^ (99% lymphocytes), protein 0.21 g/L, sugar 0.62 g/L	IVIG, ASA, steroids	No	Full recovery after 3 d of treatment	Small left coronary artery (*Z* score 4) and right coronary artery (*Z* score 3) aneurysms at follow-up 6 mo after admission
Rodriguez-Gonzalez et al^[29]^	1	4	F	L	7	9	Before	Left CAA (4.4 mm; *Z* score 9.51), proximal right CAA (4.2 mm; *Z* score 9.51) and CA ectasia at all segments, saccular aneurysm in proximal right CA	In the second week	Before	Yes	Normal	IVIG, ASA, enalapril, subcutaneous enoxaparin	No	Full recovery after 7 d of treatment	Recovery of CAAs and minimal improvement of CA ectasia after 3 mo
Yuan and Lu^[30]^	1	3	M	L	8	8	Before	Left CA dilatation (3.06 mm; *Z* score 4.98)	8 d	Before	Yes	Normal	IVIG, ASA	No	Full recovery within 2 d after onset	Complete recovery after 6 mo
Zhang et al^[31]^	1	6	M	Bilateral	L 6; R 9	In the third week	Before	Bilateral CAAs: left 5.5 mm, right 6.2 mm	14 d	Before	Yes	WBC 36/mm^3^ (97% mononuclear cells), normal protein and sugar, negative culture	IVIG, ASA	No	Right: resolution after 1 mo of treatment; Left: persistence during the 18-mo follow-up	Persistent dilatation of CAs (left 4 mm, right 5.2 mm)
Yu et al^[32]^	1	7	F	Bilateral	L 14; R 17	19	Before	Beaded sample dilatation of all CAs, in addition to aneurysms of the middle of the right CA (6.2 mm, *Z* score 14.5) and left CA (5.4 mm, *Z* score 9.4)	In the third week	Before	No	WBC 36/mm^3^, normal protein and sugar	IVIG, ASA, warfarin	No	Partial improvement on the left and complete resolution on the right after 2.5 yrs	Persistent dilatation of CAs (right 3.6 mm, 5.2 *Z* score; left 6.5 mm, 9.0 *Z* score, mural thrombus) after 2.5 yrs
Yuan and Lu^[33]^	1	4	F	R	7	10	Before	Aneurysms of the right main CA (maximum diameter of 8.5 mm; *Z* score 11.62), left main CA (maximum diameter of 5.1 mm; *Z* score 6.97), left anterior descending CA (maximum diameter of 3.3 mm; *Z* score 4.26), and left circumflex CA (maximum diameter of 2.2 mm; *Z* score 3.01), mild pericardial effusion, mitral regurgitation	In the second week	Before	Yes	Normal	IVIG, ASA, enoxaparin, warfarin	No	Full recovery at the 3-mo follow-up after discharge	Improvement in the right CA aneurysms, left CA and left anterior descending CA, normal left circumflex CA, disappearance of pericardial effusion and mitral regurgitation at the 3-mo follow-up after discharge
Peña-Juárez et al^[34]^	1	9	F	L	29	9	After	Bilateral giant CAAs	In the fourth week	After	No	¯	IVIG (4 doses), ASA, steroids, infliximab, cyclosporine, acenocoumarin	Yes	Recovery after 1 wk	Partial recovery after one-year follow-up
Chen et al^[35]^	9	8; 3; 8; 13; 4; 24; 5; 108;15	5 M; 4 F	6 L; 2 R; 1 Bilateral	6; 6; 10; 16; 10; 11; 8; 15 + 4; 11	9; 10; 6; 19; 14; 7; 11; 9; 6	5 Before; 4 After	In 8 out of 9 patients: CAA in 4 cases and CA dilatation in 4 cases	¯	¯	3 Yes; 6 No	In 6 out of 9 patients: normal in 4 cases; WBC 12/mm^3^, protein 0.451 g/L in one case; WBC 5/mm^3^, protein 0.4153 g/L in one case	IVIG, ASA，short-term dexamethasone	No	27; 57; 10; 15;11; 25; 36; 19; 130 d	16; 12; 87; 14; 40; 12; 282 d
Adachi et al^[36]^	1	18	F	L	11	4	After	None	None	None	No	¯	IVIG, prednisolone	No	FNP improvement after 4 mo; persistent unrecovered synkinesis of left eyelids and mouth	None
Maglione et al^[37]^	1	4	M	R	6	<10	Before	Aneurysms of the right CA (4 mm, *Z* score 9) and the proximal right CA (3.3 mm; *Z* score 6); dilatation of the left CA (2.8 mm; *Z* score 3.9)	7 d	Before	Yes	¯	IVIG, ASA, steroids, clopidogrel	No	Full recovery after 1 mo	Partial improvement after 4 mo
Murata et al^[38]^	1	4	F	L	10	>12	Before	Right and left CALs (*Z* score 11.1 and 4.07)	In the second week	Before	Yes	¯	IVIG, prednisolone, mecobalamin	No	Complete improvement after 6 wks	Partial improvement after 2 yrs
Current report	1	4	M	Bilateral	L 8; R 13	19	Before	Right main CA dilatation (2.7 mm; *Z* score 4.06)	14 d	Before	No	Not done	IVIG, ASA, dexamethasone	No	L: 6 d; R: 4 d	Complete recovery after 5 d

“–” = not mentioned in the literature, ASA = acetyl salicylic acid, CA = coronary artery, CAA = coronary artery aneurysm, CALs = coronary artery lesions, CSF = cerebrospinal fluid, FNP = facial nerve palsy, iKD = incomplete Kawasaki disease, IVIG = intravenous immunoglobulin, KD = Kawasaki disease, WBC = white blood count.

FNP is a rare complication of KD, published mainly in case reports worldwide. The attending doctor missed the diagnosis of incomplete KD (iKD) on the first admission because of atypical features (spontaneous fever remission and transient bilateral conjunctival injection). Therefore, the differential diagnosis of FNP is of great significance. In the etiology, trauma, idiopathic, infectious, neurovascular, autoimmune, inherited, congenital, toxic, iatrogenic, metabolic, and other causes can be observed. In addition to FNP, most diseases exhibit accompanying symptoms.^[39,40]^ Thorough history-taking, physical examination, biochemical analysis, and radiological imaging are essential in evaluating the potential cause of FNP. Fever and elevated inflammatory markers may suggest bacterial infection or autoimmune diseases that cause vascular inflammation and ischemia as the underlying etiologies. Bacterial meningitis should be differentiated in terms of age and clinical symptoms. However, given the patient’s good general condition, lack of any additional neurological symptoms or signs, and the normalization of temperature with antibiotics that cannot cross the blood–brain barrier on initial admission, suggesting a lower probability of bacterial meningitis, a lumbar puncture was not initially performed. Furthermore, MRA or other vascular imaging for CNS vascular involvement was necessary for further assessment of the underlying causes, such as autoimmune diseases, in the case of recurrent FNP during the second admission. Until he experienced a resurgence of fever and bilateral conjunctival injection, we made a diagnosis of complete KD because of redness at the site of BCG inoculation, reddening of lips, and desquamation of the distal extremities, and then overlooked the imaging evaluation for CNS vascular involvement during the second admission. Nevertheless, the lack of CSF analysis for meningitis and vascular imaging for CNS vascular involvement constituted limitations in our clinical practice, as they are crucial diagnostic tests that should be meticulously considered in similar clinical scenarios to differentiate from other underlying causes, as seen in our patient.

According to a report from the 24th Nationwide Surveillance in Japan, the prevalence of iKD is approximately 20% of new-onset cases, and there is a higher percentage of children aged ≤ 2 years and children aged ≥ 6 years. Nonsuppurative cervical lymphadenopathy in older children and BCG redness in infants are particularly indicative of iKD.^[41]^ Our literature analysis revealed that 42.2% of patients had iKD, all patients had fever, and the 2 most deficient criteria were changes in the peripheral extremities and nonsuppurative cervical lymphadenopathy. Of the patients, 92.9% were aged ≤ 2 years and 66.1% were aged < 12 months (Table 1). Because of the younger age distribution, delayed diagnosis and treatment of KD will be possible when FNP complicates KD. However, the age of pediatric idiopathic FNP is much older, ranging from 7 to 17 (mean, 14.7 ± 2.5) years reported by Aysel et al.^[42]^ Besides, fever is generally not a common accompanying symptom in patients with idiopathic FNP. Therefore, if a patient aged ≤ 2 years suffers from FNP, accompanied by a fever lasting for ≥ 5 days or irregular fever, KD should be on high alert.

KD can be challenging to diagnose when the initial symptoms are atypical, particularly in the context of corticosteroid administration for FNP, as observed in our patient. The family had concerns regarding the etiology, diagnosis, treatment, and outcomes because of the recurrence of the illness and considered transferring to another hospital during the second hospitalization. We conducted extensive communication with the family to address their concerns and restore their trust. Hence, it is crucial for doctors to continuously monitor the patient’s condition and engage in comprehensive communication with the family when KD cannot be clearly diagnosed initially. Doctors should carefully investigate and examine the main clinical symptoms of KD (particularly BCG redness) and focus on several laboratory parameters, including blood/urine leukocytes, CRP, erythrocyte sedimentation rate, hemoglobin, and alanine aminotransferase, which are recommended for the diagnosis of iKD.^[41]^ Routine echocardiography is also necessary.

A substantial proportion, up to 36.8% (14/38), of patients developed FNP despite IVIG treatment, suggesting overactivated inflammation. Of the 19 patients treated with initial IVIG alone, 4 (21.1%) exhibited IVIG resistance, 2 of whom received IVIG after 10 days of illness. However, the patients treated with IVIG and corticosteroids simultaneously showed no resistance to IVIG (Table 1). Liu et al^[1]^ found that inflammatory markers levels were significantly higher in KD patients with neurological involvement, and neurological involvement had a higher risk of IVIG resistance (23.5% vs 13.7%). IVIG, together with aspirin, is currently recognized as the first-line treatment for KD. However, it seems insufficient to suppress excessive inflammation in such patients, as also observed by Stowe.^[26]^ Corticosteroids, alongside IVIG and aspirin, are recommended as the initial treatment for KD patients with a high risk of IVIG resistance.^[43]^ Therefore, adding corticosteroids to IVIG may suppress overactivated inflammation and enhance the IVIG response in KD patients with FNP. Several scoring systems for predicting IVIG resistance, such as the Egami score, the Kobayashi score, and the Sano score, seem appropriate in Japan, but are likely unsuitable in other countries.^[41]^ No predictive model has been established for the general population. Currently, a Kobayashi score of ≥5 points is more widely accepted.^[44]^

The recovery time of FNP, with varying definitions of palsy duration from literatures, ranged from 1.5 to 130 days, with a median of 20 days and an average of 31 days (Table 1). Only 3 patients had persistent FNP at 4 months, 18 months, and 2.5 years of follow-up.^[31,32,36]^ Stowe^[26]^ summarized the characteristics of 21 KD patients with FNP without the use of IVIG, and almost all patients spontaneously recovered completely within a median time of 16 days and an average time of 26 days (range: 3–90 days). Consequently, most patients have a good prognosis for FNP less affected by IVIG. Corticosteroids with prednisolone or methylprednisolone at 1 to 2 mg/kg/day for 7 to 10 days should be administered as early as possible within 72 hours for Bell patients to reduce the recovery time and promote facial nerve functional recovery.^[39]^ Our patient treated with corticosteroids for 7 to 8 days within 72 hours had a fast FNP recovery, with a duration of 6 days on the left side and 4 days on the right side, which was obviously earlier than most patients (Table 1). However, our patient was briefly in remission during his first hospitalization by using corticosteroids alone and relapsed quickly after discharge, and corticosteroids alone seemed not to be an adequate treatment. Aslani et al^[45]^ demonstrated that intravenous methylprednisolone pulse could effectively control systemic and vascular inflammation and decrease CALs without obvious side effects in KD, similar to IVIG, and could be an optional treatment for KD when IVIG is unavailable. Corticosteroids in KD have the same dose, but a longer course of 15 days, compared to Bell palsy.^[41]^ Insufficient doses or courses of corticosteroids and a lack of aspirin may contribute to the resurgence of illness in our patient without IVIG treatment on initial admission.

Worldwide, the incidence of CALs in untreated patients with KD ranges from 15% to 20%, compared to 3% to 5% in regularly treated patients.^[46]^ Chen et al^[35]^ reported that the incidence of CALs was significantly higher in KD patients with FNP than in those without FNP (88.9% vs 25.9%). Our literature analysis revealed that 80.4% (45/56) exhibited CALs, with an estimated 50% affected bilaterally. At least half of the KD patients with CALs had no IVIG or delayed IVIG treatment (>10 days after illness). This situation partly caused the elevated incidence of CALs. However, in the patients even with IVIG treatment within 10 days of illness, 81.3% (13/16) still developed CALs (Table 1). Consequently, FNP may be a risk factor for CALs.

Almost all CALs developed within the first month of illness, ranging from 5 days to 20 months, with a predominant occurrence during the second week, similar to FNP onset (Table 1). Meanwhile, other cardiovascular involvements were reported in 4 patients including mild mitral valve incompetence, axillary artery aneurysm, minimal pericardial fluid, mitral regurgitation and mural thrombus.^[18,24,32,33]^ Notably, CALs can progress over time in some cases. Regular follow-up with echocardiography holds significant importance, necessitating dynamic adjustments to the anticoagulation program. In contrast to FNP, CALs are more challenging to restore. Based on limited follow-up information, 15 patients with CALs fully recovered at different times ranging from 5 days to 7 months after CALs onset (Table 1), while others needed more time to assess potential recovery. Two patients died of cardiovascular involvement without complete recovery of the FNP.^[26]^ Our patient had the fastest CALs recovery time with a duration of 5 days.

The analysis of corticosteroids use versus no corticosteroids use for first-line treatment alongside IVIG and aspirin revealed fewer CALs in the corticosteroids group with high-risk scores or receiving longer corticosteroids treatment.^[44]^ IVIG combined with corticosteroids as the main therapy for KD can decrease the risk of developing CAA and mitigate CAA progression in KD patients at diagnosis. For KD patients who are at high risk of developing CAA, IVIG with adjunctive corticosteroids (prednisone at 2 mg/kg/day, maximum 60 mg/day, tapered over 15 days, or equivalent) is recommended as initial treatment. High-risk features for CAA are defined as an age of <6 months and a *Z* score of ≥2.5 for the right coronary or the left anterior descending artery at the first echocardiography.^[43]^ Our patient had the above high-risk features for CAA. Italian guidelines recommend a single dose of intravenous methylprednisolone pulse together with IVIG and aspirin in high-risk patients (age < 1 year, CRP > 200 mg/L, severe anemia at disease onset, liver disease, albumin < 2.5 g/dL, overt CAA, septic shock or macrophage activation syndrome).^[47]^ The low incidence of FNP in KD patients makes it difficult to systematically evaluate the risk of CAA. However, our analysis demonstrates a very high incidence of CALs despite IVIG treatment in KD patients with FNP. Therefore, adding corticosteroids to IVIG may be recommended to assist in decreasing the risk of developing CALs and alleviating CALs. Besides, corticosteroids can shorten the duration of clinical symptoms, time for laboratory parameters (CRP, erythrocyte sedimentation rate), and length of hospital day without serious complications or mortality.^[44]^ Rather than ignoring evaluation in KD patients with FNP at admission, physicians should utilize the reported high-risk features for CAA or the prediction scoring systems for IVIG resistance to make a decision if it is not initially clear whether corticosteroids are needed.

37.9% (11/29) of the patients exhibited leukocytosis with a range of 12 to 123/mm^3^ in the cerebrospinal fluid (CSF), dominated by mononuclear cells, indicating a notably high incidence of aseptic meningitis. 20.7% (6/29) had slightly elevated protein levels, ranging from 0.4153 to 0.71 g/L. Only 2 patients exhibited a slight decrease in glucose levels (Table 1). CSF leukocytosis was observed in 15% of patients with KD with neurological involvement.^[1]^ CSF examinations were normal initially, but leukocytosis was observed in 2 patients after FNP onset.^[10,11]^ Meanwhile, irritability emerged as a common concomitant neurological symptom in reported FNP cases. Only one study reported other neurological sequelae namely hearing loss and ataxia.^[22]^ Thirty three patients underwent brain MRI/CT without positive findings, except for 2 patients with frontal atrophy or subdural effusion.^[3,6]^ Huang et al^[48]^ reported that KD patients aged < 1 year faced a higher risk of other system involvements, including aseptic meningitis, and a greater incidence of iKD and CALs compared to those aged 1 to 5 years. Consequently, distinguishing KD patients with neurological symptoms from bacterial meningitis is imperative due to atypical symptoms, particularly in infants. Hu et al reported that higher CSF glucose levels were more effective than other biomarkers in differentiating aseptic meningitis from bacterial meningitis,^[49]^ in addition to the predominance of mononuclear cells summarized by us.

## 5. Conclusion

Our case underscores the importance of promptly identifying KD when confronted with prolonged fever and FNP, particularly in patients aged ≤ 2 years whose symptoms are usually incomplete or atypical. FNP, although a rare complication of KD, is often associated with a high likelihood of aseptic meningitis and carries a favorable prognosis, seemingly unaffected by IVIG treatment. Importantly, FNP in patients with KD may serve as a risk factor for CALs, which are more challenging to resolve than FNP itself. Adding corticosteroids to IVIg may be recommended to reduce IVIG resistance, decrease the risk of developing CALs, and alleviate CALs.

## Author contributions

**Conceptualization:** Zhongyou Tan.

**Data curation:** Rong Ou.

**Formal analysis:** Rong Ou.

**Funding acquisition:** Zhongyou Tan.

**Methodology:** Rong Ou.

**Project administration:** Zhongyou Tan.

**Resources:** Rong Ou, Ling Liu.

**Software:** Rong Ou.

**Supervision:** Zhongyou Tan.

**Validation:** Zhongyou Tan.

**Writing – original draft:** Ling Liu.

**Writing – review & editing:** Rong Ou, Zhongyou Tan.
